# Supplementary material for: Impact of Nitisinone on the Cerebrospinal Fluid Metabolome of a Murine Model of Alkaptonuria
Source: Metabolites. 2022 May 25;12(6):477. doi: 10.3390/metabo12060477 (PMC9230570; doi:10.3390/metabo12060477)
Supplement: Supplementary file 1 [file metabolites-12-00477-s001.zip › metabolites-1735909-supplementary.pdf]

Supplementary Materials:

Supplementary information 1:

Chromatographic conditions

Liquid chromatography (LC) was performed on an Agilent 1290 Infinity II LC system. An Atlantis dC18 column (3.0 × 100 mm, 3 μm, Waters, UK) was maintained at 60 °C with a flow rate of 0.4 mL/min. Mobile phases were (A) water and (B) methanol both containing 5 mmol/L ammonium formate and 0.1% formic acid. The elution gradient started at 5% mobile phase B at 0–1 min, increasing linearly to 100% B by 12 min, held at 100% B until 14 min, returning to 95% A for 5 min to recondition the column. Injection volume was 1 μL.

Quadrupole time-of-flight mass spectrometry (QTOF-MS) conditions

An Agilent 6550 QTOF-MS, equipped with a dual jet stream electrospray ionisation source, was operated in 2 *GHz* mode, over the mass range of 50–1700, in negative and positive polarities. A reference mass correction solution was continually infused at a flow rate of 0.5 mL/min via an external isocratic pump (Agilent, Cheadle, UK) for constant mass correction (see Preparation of reference mass correction solution). Capillary and fragmentor voltages were 4000 V and 380 V, respectively. Desolvation gas temperature was 200 °C with flow rate at 15 L/min. The sheath gas temperature was 300 °C with flow rate at 12 L/min, and nebulizer pressure was 40 psi and nozzle voltage 1000 V. Data acquisition rate was 3 spectra/s.

Preparation of reference mass correction solution

Reference mass correction solution was prepared in 95:5 methanol:water containing 5 mmol/L purine (CAS No. 120-73-0), 100 mmol/L trifluoroacetic acid ammonium salt (TFA, CAS No. 3336-58-1) and 2.5 mmol/L hexakis(1H, 1H, 3H-tetrafluoropropoxy)phosphazine (HP-0921, CAS No. 58943-98-9) (Agilent, Cheadle, UK).

Reference ions monitored were: purine (*m*/*z* 121.0509) and HP-0921 (*m*/*z* 922.0098) (positive polarity) and TFA (*m*/*z* 112.9856), purine (*m*/*z* 119.0363) and HP-0921 (HP-0921 + formate adduct: *m*/*z* 966.0007) (negative polarity).

Supplementary Figure

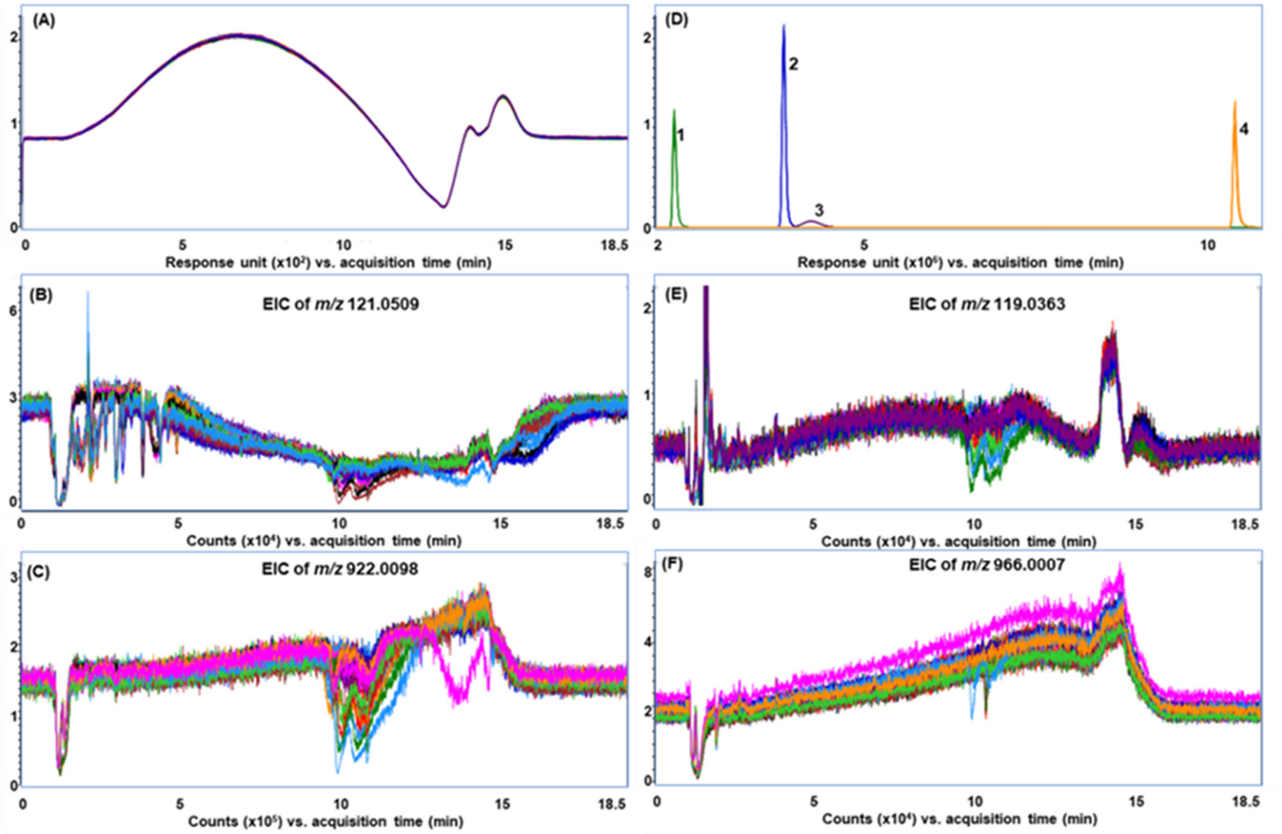

Figure S1. Summary of quality checks performed on data acquired from LC-QTOF-MS analysis of CSF samples in positive and negative polarities. (**A**) Overlaid binary pump pressure curves, (**B**) and (**C**) Overlaid reference ion signal in positive polarity; (**D**) Overlaid TIC for quality control samples analysed across the analytical sequence—1: L-tyrosine, 2—phenylalanine, 3—succinylacetone, 4—nitisinone (peaks 1, 2, and 4 positive polarity and peak 3 negative polarity); (**E**,**F**) Overlaid reference ion signal in negative polarity. Extracted ion chromatograms of reference masses were performed to check mass accuracy remained <5 ppm throughout the run. Reference ion signal is present during the analytical runs in both polarities, but shows the greatest suppression of signal at 1–1.5 min. This is near the column void volume where ion suppression is greatest due to elution of non-retained entities.
